# A multi-scale spatial model of hepatitis-B viral dynamics

**DOI:** 10.1371/journal.pone.0188209

**Published:** 2017-12-07

**Authors:** Quentin Cangelosi, Shawn A. Means, Harvey Ho

**Affiliations:** 1 Computational and Mathematical Engineering, Institut National des Sciences Appliquées, Toulouse, France; 2 Auckland Bioengineering Institute, University of Auckland, Auckland, New Zealand; Indiana University, UNITED STATES

## Abstract

Chronic hepatitis B viral infection (HBV) afflicts around 250 million individuals globally and few options for treatment exist. Once infected, the virus entrenches itself in the liver with a notoriously resilient colonisation of viral DNA (covalently-closed circular DNA, cccDNA). The majority of infections are cleared, yet we do not understand why 5% of adult immune responses fail leading to the chronic state with its collateral morbid effects such as cirrhosis and eventual hepatic carcinoma. The liver environment exhibits particularly complex spatial structures for metabolic processing and corresponding distributions of nutrients and transporters that may influence successful HBV entrenchment. We assembled a multi-scaled mathematical model of the fundamental hepatic processing unit, the sinusoid, into a whole-liver representation to investigate the impact of this intrinsic spatial heterogeneity on the HBV dynamic. Our results suggest HBV may be exploiting spatial aspects of the liver environment. We distributed increased HBV replication rates coincident with elevated levels of nutrients in the sinusoid entry point (the periportal region) in tandem with similar distributions of hepatocyte transporters key to HBV invasion (e.g., the sodium-taurocholate cotransporting polypeptide or NTCP), or immune system activity. According to our results, such co-alignment of spatial distributions may contribute to persistence of HBV infections, depending on spatial distributions and intensity of immune response as well. Moreover, inspired by previous HBV models and experimentalist suggestions of extra-hepatic HBV replication, we tested in our model influence of HBV blood replication and observe an overall nominal effect on persistent liver infection. Regardless, we confirm prior results showing a solo cccDNA is sufficient to re-infect an entire liver, with corresponding concerns for transplantation and treatment.

## Introduction

For the nearly 250 million people worldwide suffering from chronic hepatitis B virus (HBV) infection, there are few effective options for treatment [1] and its elimination is exceptionally difficult. Primary infection is maternal-neonatal vertical, but also 5% of horizontal or person-to-person [2], resulting in entrenchment of closed circularly-covalent DNA (cccDNA). cccDNA persistence and HBV resurgence (e.g., during immunosuppression for chemotherapy [3]) is associated with liver pathologies such as tissue damage (fibrosis, cirrhosis) and hepatic carcinomas [4]. Current treatments are mitigatory and require sustained and prolonged protocols (e.g., reverse transcriptase inhibitors) but without cccDNA eradication leading to reactivation upon cessation [5]. cccDNA entrenchment further evades detection complicating transfusions or transplantations [6].

Currently we do not have a clear characterisation of the HBV-immune dynamic and why 5% of immune responses in adults fail [7] [8]. Innate immune activity, e.g. natural killer (NK) cells and resident liver macrophages (Kupffer cells), and its more generalised recognition of pathogens possibly permits stealth infections of HBV [9] or is a critical guardian against successful HBV colonisation [7]. Meanwhile, adaptive responses (e.g., CD4+/CD8+ T-Cells) and their specificity are possibly essential for confining infection to the 95% of acute cases, yet is also suspect for cytolytic tissue damage [10].

Moreover, mutually disruptive innate and adaptive immune activity occurs. Key to HBV invasion is the sodium-taurocholate cotransporting polypeptide (NTCP) [11], a promising target for blocking HBV uptake, yet its downregulation with interleukin-6 blocks apoptotic mechanisms and may even assist HBV progress [12]. Targeting of cccDNA with interferon-alpha and lymphotoxin-beta further unexpectedly stimulates NK cells to terminate adaptive CD8+ T-cells [13].

Previous mathematical modeling efforts shed considerable light on the HBV dynamic. Murray, et al. fitted chimpanzee model data from the Chisari laboratory and determined CD8+ T-cells induce a non-cytopathic blockage of viral RNA conversion to DNA-containing capsids, further leading to removal of cccDNA during clearance of an acute HBV infection [14]. Ciupe, et al. [15] tested experimentalist suggestions that hepatocytes generated during replenishment after immune cytolysis are refractory to HBV infection [16]. The precise mechanisms characteristic of a refractory cell are unclear, perhaps involving successful suppression of HBV viral replication through cytokines or a lack of NTCP expression in novel hepatocytes. Regardless, such refractory mechanisms permitted the Ciupe, et al. model to fit given patient data and was consistent with later experimental investigations, yet with some caveats. Mason et al. showed in animal models that cytokine suppression of HBV replication during the immune clearance phase is incomplete, and leads to large hepatocyte turnovers of around 2.2 to 4.8 times more than necessary for viral clearance [17]. Further, they suggested successful immune clearance of HBV from the liver occurs via scenarios where cccDNA fails to survive mitosis during generation of new hepatocytes. Such scenarios were also suggested by the mathematical results from Murray & Goyal [18] where they observed clearance of HBV without emergence of refractory cells—as long as HBV viral components are lost during cell proliferation. We thus consider an alternative to emergence of refractory mechanisms for failure to clear HBV.

As a further complication, transplantation of pristine uninfected livers often leads to rapid HBV reinfection [19]; this inspired inference of extra-hepatic HBV reservoirs as in the Qesmi, et al. mathematical model [20]. Their analysis indicated that existence of such extra-hepatic reservoirs condemn treatments of the liver alone to failure without therapies including testing for viral activity both inside and out of the liver. HBV is indeed observed in extra-hepatic tissues such as the pancreas and kidney—organs expressing the NTCP [21]—as well as the skin [22] and these extra-hepatic infections may provide a reservoir of viable and infective HBV for invasion of pristine liver transplants. Some suggest not only residence of HBV in extra-hepatic tissues but also active replication. Mason, et al. [23] detected HBV viral replication intermediates in a variety of extra-hepatic tissues in individuals chronically infected with HBV, and more recent observations of replication in lymphatic tissues, bone marrow and peripheral blood mononuclear cells (PBMCs) support the assertion [24]. The significance of these extra-hepatic replication sites is unclear, and whether they provide a substantial reservoir of actively replicating and infective HBV awaits confirmation. Such extra-hepatic infection and replication may explain some associated extra-hepatic complications of HBV (e.g., dermatologic diseases, arthritis and neuropathy [17]), however claims of extra-hepatic HBV replication remains controversial.

Hepatic environments are intrinsically spatially heterogeneous, where hepatic lobe structure is classically divided per oxygen- and nutrient-rich zones according to proximity with the portal triad and metabolic function [25]. Densities of the hepatic macrophage Kupffer cells are weighted heavily around the portal triad inlets [26], putatively as protective filters of antigen flows into the hepatic matrix. Eventual fibrosis compromises adaptive T-cell monitoring of hepatocytes by defenestration of sinusoidal epithelia blocking surface antigen surveillance and cytolytic response [7]. *In silico* modeling of hepatic drug perfusion further demonstrates a subtle impact of spatial distributions for hepatocyte steatosis [27]. These variant physical and spatial components may be crucial to the establishment of chronic HBV such as through variations in HBV uptake via the NTCP, immune-cell activity, or the intracellular HBV replication cycle itself due to, say, nutrient concentration near the periportal inlet. We hypothesise that well-known zonations of lobe structure might correspond to spatial concentrations of HBV activity thus providing a reservoir for infection. Our literature searches on any spatial components regarding HBV return limited if any results. We are not aware of any published results regarding experimental observations of spatially-localised HBV expression. Yet HBV replication was noted exploiting similar transduction pathways to gluconeogensis [28] that has known elevated activity in periportal regions [25], thus suggesting a spatial element to HBV. No investigations of HBV within the context of these spatial aspects currently exist, however, either with animal or mathematical models. In order to investigate our hypothesis, we present a multi-scale mathematical modeling scheme for determining under what spatial distributions persistence of infection arises—with some additional attention to the controversial and complicating extra-hepatic replication of HBV. Our overall aim is determining whether such spatial aspects may indeed be worthy of additional experimental attention.

## Materials and methods

### Prior mathematical models

Deterministic, or non-stochastic methods (ordinary differential equations) that provide approximations to the average numbers of virions, typically treat the liver as a spatially homogeneous compartment with three coupled elements: pristine or uninfected hepatocytes, infected hepatocytes and virus, such as the classic Nowak, et al. model [29]. They performed a traditional analysis of conditions producing an endemic infection (*R*_0_ > 1) and showed drug efficacy was key to driving *R*_0_ below 1 to eliminate the infection. The series of models from Ciupe, et al. added immune cell dynamics and the emergence of refractory elements [15], or antibody & viral complexes [30] with some focus on roles of viral antigens on prenatal immunotolerance [31]. Their results showed refractory cell emergence was necessary to fit available data, or that a large amount of antibodies forming complexes with HBV antigens could have a similar effect. Alternatively, we know of only the Qesmi, et al. model [20] that includes an additional compartment for the blood, with blood cells susceptible to infection by and replication of HBV. Their addition and subsequent analysis indicated no therapy aimed solely at liver infection could succeed in HBV eradication and any treatment must be accompanied by testing for viral production in extra-hepatic environments as well. None of the deterministic models we surveyed included any spatial representation of liver structure.

The stochastic models of Murray & Goyal are based on deterministic methods but provide approximations to the statistical distributions of virions. They utilise stochastic distributions (Poisson) around the averaged solution provided by the deterministic ordinary differential equations in agent-based implementations. With their intracellular HBV infection model including uptake via NTCP, conversion to cccDNA, expression as protein (one of the three HBsAg) and release—described below in Methods—they include some spatial aspects as in their recent work [32]. Aimed at testing influence of cell-cell transmission (CCT) of HBV where transport of viral particles directly infected neighbouring cells distributed on a lattice, they suggested CCT is not essential to establishing infection but nevertheless hinders immune-clearance. Their prior model [18] foundational to the CCT investigation further showed refractory cells are not necessary to viral clearance. Notably, neither of their models mentioned here include spatial considerations such as distribution of nutrients, blood transport or any extra-hepatic compartment.

**Fig 1:**
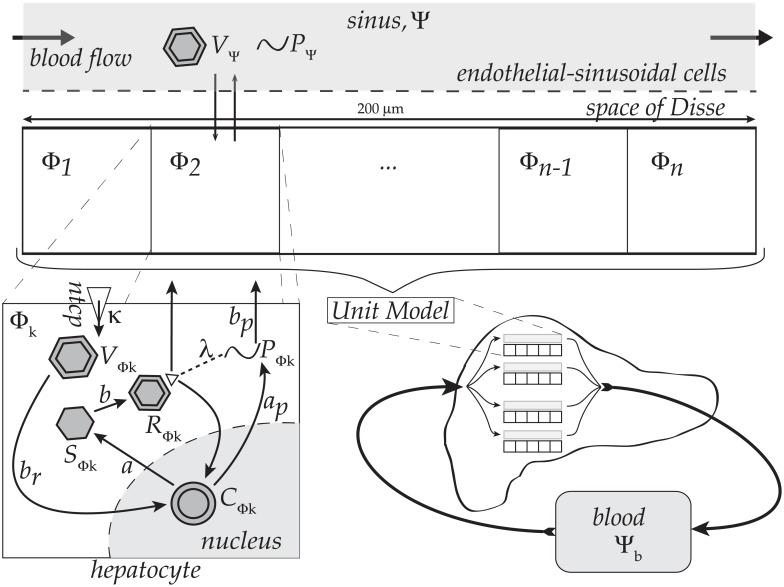
Diagram of the multiscale modeling framework. Assembly of the multi-scaled model including *k* = 1…*n* intracellular hepatocyte HBV models (Φ_*k*_) coupled to a sinusoidal lumen (Ψ) and extra-hepatic blood compartments (Ψ_*b*_). The original Murray-Goyal model was for a single hepatocyte; we here expand their single-hepatocyte model across *n* unique hepatocytes with *n* intracellular HBV models (Φ_*k*_). Thus, the *k*^*th*^ hepatocyte expresses a unique number of viral particles as determined by its own respective *k*^*th*^ intracellular compartmental model. Within the *k* = 1…*n* hepatocytes, the HBV model functions as follows. Complete viral particles (*V*_Ψ_) taken into the cell, denoted *V*_Φ*k*_, then convert to cccDNA in the nucleus (*C*_Φ*k*_) which in turn trigger production of viral proteins p36 (*P*_Φ*k*_) and ‘single-strand’ viral DNA (*S*_Φ*k*_). *S*_Φ*k*_ is in turn converted to ‘relaxed’ viral DNA (*R*_Φ*k*_) that either returns to the nucleus reinforcing the cccDNA population (*C*_Φ*k*_) or is released from the cell to *V*_Ψ_. The amount of *P*_Φ*k*_ in the cell determines which route *R*_Φ*k*_ will take, and *P*_Φ*k*_ itself is also released into Ψ as *P*_Ψ_. Note release of complete viral particles from the *k*^*th*^ hepatocyte (e.g., *V*_Φ*k*_ and *P*_Φ*k*_) is added to the total pool of sinusoidal lumen viral particles (e.g., *V*_Ψ_ and *P*_Ψ_), at the respective spatial location for the *k*^*th*^ hepatocyte. Hence, the suite of *n* hepatocytes are effectively coupled by viral transport to and from the sinusoid lumen (Ψ). No other coupling between the hepatocytes occurs in our model (e.g., gap-junctions, etc.) An additional compartment representing the extra-hepatic blood, Ψ_*b*_, includes dynamics of infection for red blood cells as noted in the text.

The multi-scaled mathematical framework of HBV infection dynamics we assemble here includes building blocks, adopted from several models reflecting different spatial scales, as illustrated in Fig 1. At the organ and sinusoidal level, the scheme is inspired by a method proposed [27] aimed at capturing spatial heterogeneities via representative sinusoids, which are rows of hepatocytes flanking blood vessels or sinuses. At the intra-cellular level, we incorporate the agent-based and thus stochastic HBV dynamic model of Murray & Goyal mentioned above for each hepatocyte. Each of these representative sinusoids we label as our ‘unit model’. A suite of unit models are then assembled to sample activity over the liver including micro-scaled spatial heterogeneities without modeling the entire hepatocyte population. At the organism scale, the Qesmi, et al. model [20] is used where the suite of unit models are coupled to a blood compartment including another extra-hepatic HBV replication model. We disable or enable the extra-hepatic replication component as noted throughout the text for comparison. For complete details on each component model we utilise in our spatial representative assembly, refer to the original references for a thorough introduction to their respective work instead of our brief summation here.

### Hepatocyte-sinusoid HBV model

#### Mathematical representation

The model deployed here is an agent-based formulation extended across suites of hepatocytes derived from the time-delayed differential equations described in [18] but with modifications to account for the HBV exchange between a hepatocyte and the sinusoidal lumen. The deterministic equations here are varied stochastically according the agent-based scheme described below and is taken directly from [18]. The model contains two primary mechanisms, the sinusoidal HBV dynamic model, situated in compartment Ψ, and the *k*^*th*^ hepatocyte HBV dynamic model Φ_*k*_:

$$\begin{array}{l} \Psi :\\ \{\begin{array}{l} \frac{dV_{\Psi }}{dt}=J_{V_{\Psi }}^{+}\left(P_{\Phi },R_{\Phi k}\right)-J_{V_{\Psi }}^{-}\left(V_{\Psi }\right)-cV_{\Psi }\\ \frac{dP_{\Psi }}{dt}=J_{P_{\Psi }}^{+}\left(P_{\Phi }\right)-cP_{\Psi } \end{array} \end{array}$$ (1)

$$\begin{array}{c} \begin{array}{c} \Phi_{k}:\\ \left\{ \begin{array}{cc} \frac{dV_{\Phi }}{dt} & =J_{V_{\Psi }}^{-}\left(V_{\Psi }\right)-b_{r}e^{\left(-\lambda P_{\Phi }\right)}V_{\Phi }-\mu_{r}V_{\Phi }\\ \frac{dC_{\Phi }}{dt} & =be^{\left(-\lambda P_{\Phi }\left(t-\tau \right)\right)}\left[R_{\Phi }\left(t-\tau \right)+V_{\Phi }\left(t-\tau \right)\right]-\mu C_{\Phi }\\ \frac{dS_{\Phi }}{dt} & =aC_{\Phi }\left(t-\tau \right)-bS_{\Phi }\\ \frac{dR_{\Phi }}{dt} & =bS_{\Phi }\left(t-\tau \right)-bR_{\Phi }\\ \frac{dP_{\Phi }}{dt} & =a_{P}C_{\Phi }\left(t-\tau \right)-J_{P_{\Psi }}^{+}\left(P_{\Phi }\right). \end{array}\right. \end{array} \end{array}$$ (2)

Each dynamic variable is as described in Table 1 for tracking lumenal HBV and protein (*V*_Ψ_ and *P*_Ψ_, respectively) and intracellular viral particles, both whole virions and component (*V*_Φ_, *C*_Φ_, *S*_Φ_, *R*_Φ_) and protein (*P*_Φ_) for the *k*^*th*^ hepatocyte. The protein particle variable for *p*36 in our model is identical to those in Murray & Goyal, and represents the largest of three proteins comprising the HBV surface antigen, or HBsAg. Note for simplicity here we omit the index *k* for intracellular particles. We introduce boundary flux terms $J_{V_{\Psi }}^{-}\left(V_{\Psi }\right)$ for *V*_Ψ_ and $J_{P_{\Psi }}^{+}\left(P_{\Phi }\right)$ for *P*_Φ_ that are distinct from the original Murray & Goyal model since we include spatial components in our implementation. We do include the same decay rates (*c*) in the sinus compartment, Ψ, for *V*_Ψ_ and *P*_Ψ_. Essentially, each *J* flux term is simply a boundary transport version of the same reaction term in [18] but described as flux in our spatial heterogeneity model with the following necessary and distinct formulation:

$$\begin{array}{c} \begin{array}{cc} J_{V_{\Psi }}^{+}\left(P_{\Phi },R_{\Phi }\right) & =b\left(1-e^{\left(-\lambda P_{\Phi }\left(t-\tau \right)\right)}\right)R_{\Phi }\left(t-\tau \right)\\ J_{V_{\Psi }}^{-}\left(V_{\Psi }\right) & =\kappa V_{\Psi }\left(t-\tau \right)\\ J_{P_{\Psi }}^{+}\left(P_{\Phi }\right) & =b_{p}P_{\Phi }\left(t-\tau \right). \end{array} \end{array}$$ (3)

The parameter, *τ*, represents the time delays throughout the intracellular model components; e.g., conversion of *V*_Φ*k*_ to *C*_Φ*k*_, production of *P*_Φ*k*_, and the flux $J_{V_{\Psi }}^{+}\left(P_{\Phi k},R_{\Phi k}\right)$. This is precisely as taken from the Murray & Goyal model; see [18] for details on justification for this delay term, *τ*. We also follow Murray & Goyal by including probabilistic influence of *P*_Φ*k*_ on whether *R*_Φ*k*_ returns to the nucleus to join *C*_Φ*k*_ (exponential term in *dC*_Φ_/*dt*) or is released to the *V*_Ψ_ population (via $J_{V_{\Psi }}^{+}$), as suggested by observations [33]. Of note, the production rate for *P*_Φ*k*_ (*a*_*p*_) is 1000-fold higher than for single-strand HBV DNA (*S*_Φ*k*_) since HBV surface antigens (HBsAg) levels are approximately 1000-fold higher than particle counts for *S*_Φ*k*_. See model schematic in Fig 1, associated parameter details in Table 2, and see Murray & Goyal for further details on the parameters for their model we utilise here as noted in the Table of parameters [18].

**Table 1:** Table of notations.

Notation	Item
Ψ	Sinusoidal Lumen
Φ_*k*_	*k*^*th*^ Hepatocyte cell (cytosolic)
*V*_Ψ_	Virion in Ψ (sinusoidal HBV)
*P*_Ψ_	p36 protein in Ψ
*V*_Φ*k*_	Virion in *k*^*th*^ hepatocyte cell (cytosolic HBV)
*P*_Φ*k*_	p36 protein in *k*^*th*^ hepatocyte
*C*_Φ*k*_	cccDNA in *k*^*th*^ hepatocyte
*S*_Φ*k*_	single-stranded DNA in *k*^*th*^ hepatocyte
*R*_Φ*k*_	‘relaxed’ DNA in *k*^*th*^ hepatocyte (also cytosolic HBV)
Γ	Vector of HBV replication parameters (*b*_*r*_, *a*, *a*_*p*_,…)
Ψ_*b*_	Extra-hepatic blood

*V*_Ψ_, *V*_Φ*k*_ and *R*_Φ*k*_ are all the same complete Hepatitis B viral particle and so will also be named HBV, where subscript ‘Φ*k*’ indicates cytosolic particles in the *k*^*th*^ hepatocyte, and ‘Ψ’ indicates particles in the sinusoidal lumen.

**Table 2:** Table of parameters.

Name	Role	Value ($\frac{1}{day}$)	Ref
*a*	*C*_Φ*k*_ → *S*_Φ*k*_ conversion rate	50	[18]
*a*_*p*_	*P*_Φ*k*_ production rate	1000 × a	[18]
*b*	*S*_Φ*k*_ → *R*_Φ*k*_ & *R*_Φ*k*_ → *C*_Φ*k*_ or *V*_Ψ_ rate	log(2)	[18]
*b*_*p*_	*P*_Φ*k*_ to *P*_Ψ_ transport rate	*b*	[18]
*b*_*r*_	*V*_Φ*k*_ → *C*_Φ*k*_ conversion rate	*b*	[18]
*κ*	*V*_Ψ_ → *V*_Φ*k*_ transport rate	0.3	[18]
*μ*_*r*_	*V*_Φ*k*_ degradation rate	log(2)	[18]
*c*	*P*_Ψ_ & *V*_Ψ_ degradation rate	24log(2)/4	[18]
*μ*	*C*_Φ*k*_ degradation rate	log(2)/50	[18]
λ	*P*_Φ*k*_ influence on *R*_Φ*k*_ → *V*_Ψ_ or *C*_Φ*k*_	1/100000	[18]
*γ*	Model: liver hepatocyte ratio	150: 6 × 10^10^	Calculated
λ_*z*_	Blood cell births	10^5^ cells	[20]
*d*_*z*_	Blood cell deaths	0.03 cells	[20]
*β*_*z*_	Blood cell infectivity	2 × 10^−10^/*virion*	[20]
*δ*	Cytolytic Response	0.3, 0.6, 0.9	Varied [18]
*u*	Non-Cytolytic Response (dimless)	0.25, 0.5, 0.75	Varied [18]

As detailed in the text, these parameters are included in Eqs (1) and (2) as depicted by Fig 1). Values are taken from citations given, varied as noted, or calculated as necessary. All parameters are dimensions 1/*day* except non-cytolytic immune response, *u*, which is dimensionless. See text or cited references for more description of these items.

Hepatocyte life-span being 180 days [34], we modeled natural death by removing the *k*^*th*^ hepatocyte via a random selection following a binary ‘coin-flipping’ method otherwise known as a Bernoulli process, $B(\frac{\Delta t}{180})$, at each time step Δ*t* (in days). We then assume that a compensatory proliferation of cells permit the liver to keep the same population size of hepatocytes. New hepatocytes are added at the same rate they are removed keeping a constant number of cells at any time. Survival of HBV particles, such as DNA strands, *S*_Φ*k*_, *R*_Φ*k*_, or the protein *P*_Φ*k*_, through to daughter cells was considered by Murray & Goyal. Their results suggested that viral loss must be quite high during cellular division in order to achieve observed levels of hepatocyte turnover (HT) [18] (see below section Immune Response). Of particular concern is the propagation of tenacious cccDNA through mitosis that is observed to varying degrees in duck, woodchuck and human infections. Comparisons between control groups and those treated with anti-virals, Mason, et al. proposed cccDNA does not survive mitosis in HBV-infected woodchucks [35]. Reaiche, et al. further observed for duck HBV substantial survival of cccDNA in some instances, but in others loss of all cccDNA [36]. More recent work by Allweiss, et al. on human HBV found hepatocyte proliferation potently destabilises cccDNA, suggesting clearance of cccDNA in the great majority of cells [37]. Consistent with the vast majority of cccDNA and apparently other viral particles failing to survive mitosis, we model replenishment of hepatocytes as pristine. However, we vary the level of cccDNA survival for comparison by testing survival rates of 2% and note as such in the text. Notably, we do not include any refractory aspect for these pristine hepatocytes.

#### Immune response

Immune response after infection ideally eliminates hepatitis B viral particles. There are several types of immune actions from the adaptive and the innate ranging from disruption of intracellular viral activity to cytolytic elimination of infected host cells. Although there are myriad immune actions that are relevant, such as antibodies neutralising the virus, we only include the two actions described as the cytolytic (CYL) and non-CYL as utilised in the Murray & Goyal implementation, and represent them in the following manner [18]. Note, we do not intensively focus on the subtle interplay between these two immune responses due to our interest in the influence of spatial distributions; rather, see the Murray & Goyal model for such an investigation at the cellular level.

CYL action consists in killing infected cells with a rate related to the strength of immune response. This is modeled by a probability of $1-exp(-\frac{\delta \Delta t}{1+I/N})$ to remove the infected hepatocyte where *δ* is the strength of immune response (in *day*^−1^) and *I* is the number of infected cells. *N* is the total number of cells—that we keep fixed with the replenishment scheme described above. We assume the probability of CYL killing is lower as number of infected cells increases, essentially reflecting the limited capacity of CYL immune action. Our CYL activity as a function of this rate constant, *δ*, is virtually identical to that used by Murray & Goyal with a subtle difference. Murray & Goyal determine probability that a set of infected cells to be destroyed, whereas we compute a probability that an individual infected cell is destroyed. The probability of CYL action on any infected individual hepatocyte is then indirectly influenced through the strength rate parameter, *δ*.

The cytolytic action represented via *δ* further influences the HT for our virtual liver. Our implementation of the Murray & Goyal model here naturally follows their HT levels; we briefly present their calculation here. Considering infected (*I*) and healthy hepatocytes (*H*), removal of infected (*δI*) and their dynamic described by:

$$\frac{dI}{dt}=-\delta I(1+I/H)$$ (4)

$$\delta I=\frac{dI/dt}{1+I/H},$$ (5)

$$\Rightarrow \int_{0}^{\infty }\delta Idt=\int_{0}^{H}\frac{dI}{1+I/H}=Hln\left(2\right).$$ (6)

We can of course vary this level of turnover through the CYL immune parameter, *δ*, and we restrict our overall use of *δ* to ranges less than 1.0 resulting in turnover levels at or below the maximum observed range (e.g., 0.7–1.0, [38]) unless otherwise noted. See [18] for more detail on their discussion of this aspect.

Non-CYL immune response, instead of outright killing the infected cell, merely inhibits intracellular activity of the virus. For instance, production of single-stranded DNA (ssDNA or *S*_Φ*k*_) is inhibited by alpha/beta interferons disrupting formation of pregenomic RNA and hence its transcription into ssDNA (*S*_Φ*k*_) [14] [39]. In our model, we represent this strength of non-CYL immune response with the term *u*, where the viral *S*_Φ*k*_ production rate *a* is reduced by a factor 1−*u*. Hence in our model, maximal non-CYL immune intervention occurs at *u* = 1.

### HBV circulation-through-liver model

Transport of HBV in the sinus (region Ψ, Fig 1) is represented with the following advection-reaction scheme. We change the notations of reaction operators from the ones given in the system of Eqs (1 and 2), to separate the reaction (*ψ*, *φ*) and flux terms (*J*), for a simplified overview of the model thus:

$$\begin{array}{c} \begin{array}{cc} \frac{\partial \vec{v_{\Psi }}}{\partial t} & =\nu \frac{\partial \vec{v_{\Psi }}}{\partial x}-\sum_{k}\;J^{k}\left(\vec{v_{\Psi }},\vec{v_{k}}\right)+\psi \left(\vec{v_{\Psi }}\right)\\ \frac{d\vec{v_{k}}}{dt} & =J^{k}\left(\vec{v_{\Psi }},\vec{v_{k}}\right)+\varphi^{k}\left(\vec{v_{k}}\right). \end{array} \end{array}$$ (7)

For compactness we define $\vec{v_{\Psi }}$ and $\vec{v_{k}}$ as vectors of $R^{2}$ and $R^{5}$ gathering all particles from the sinusoid model, Eq (1) and *k*^*th*^ hepatocyte model, Eq (2), respectively. $J^{k}\left(\vec{v_{\Psi }},\vec{v_{k}}\right)$ is transport of viral particles between the sinusoidal lumen Ψ and the *k*^*th*^ hepatocyte (Φ_*k*_). *ψ*(*v*_Ψ_) are reactions from the sinusoidal HBV dynamic model, Eq (1), and *φ*^*k*^(*v*_*k*_) are reactions from the intracellular HBV dynamics model, Eq (2). Advective transport along *x* is one-dimensional and runs the length of the sinusoid with 30 hepatocytes spanning 200 *μm*.

Flow rate or velocity *ν* is estimated as follows. The liver on average weighs around 1.3 kg with a volume of roughly 1.5 L and contains about 13% of all blood flowing through the body at any one time. Two inflows directing blood into the liver are the portal vein (PV) and the hepatic artery (HA), with flow estimates ranging from 64 to 84 L / hour [40] [41], resulting in an upper level for complete renewal of liver blood supply at every 64 seconds. This is a comparable to the 1.47 L / min computed in Chalhoub, et al. [42] from a sinusoidal flow rate of 183 *μm*/*s*—a rather high fluid velocity through our representative sinusoids. These multiple time scales for blood transport and HBV dynamics are discussed in Solution Methods below.

The suite of 5 sinusoids in our representative live model are all treated as parallel components; no direct coupling of blood between them occurs in our model. Instead, they are indirectly coupled through the extra-hepatic blood compartment, Ψ_*b*_. Blood inflow through the PV and the HA is divided equally amongst the 5 sinusoids as well as exits from the suite of sinusoids back to the blood. Particle counts of HBV in the blood (e.g., *V*_Ψ_ and *P*_Ψ_) are subdivided equally—as much as possible—between the 5 sinusoidal blood inlets as well, and particle counts combined as they exit back to the blood compartment.

### Extra-hepatic HBV model (Region Ψ_*b*_)

HBV infections are observed in extra-hepatic tissues [22] as well as replicative intermediates suggesting active HBV production [23]. Despite these observations it remains controversial whether such extra-hepatic infections lead to viable and infective virions. Typically virus infections are organotrophic in nature and HBV is considered no exception. The observations, however, of HBV reinfection of otherwise pristine liver transplantations [19] inspired introduction of an extrahepatic HBV model including replication to analyse the implications of such an extrahepatic HBV reservoir. The Qesmi, et al. model [20] introduced such a model for the blood, and we borrow from their formulation with some modifications. For our purposes, though, we utilise the Qesmi, et al. blood HBV model as representative of HBV activity in all extra-hepatic tissues. This is of course a simplification: the myriad tissue and cell types observed with HBV will likely vary in uptake, replication and release rates. For simplicity however, due to our primary focus on impact of spatial heterogeneity, we deploy the Qesmi, et al. scheme here primarily to observe the effect of such extra-hepatic replication on clearance of HBV.

For the blood compartment in our model, Ψ_*b*_, we consider HBV circulating through the human body as in a closed circuit. After flowing through the liver, blood is transported to the rest of the body which we simply represent as a second, well-mixed compartment:

$$\begin{array}{c} \begin{array}{c} \Psi_{b}:\\ \left\{ \begin{array}{cc} \frac{dz}{dt} & =\lambda_{z}-\beta_{z}zV_{b}-d_{z}z\\ \frac{dV_{b}}{dt} & =k_{z}w-c_{2}V_{b}-\beta_{z}zV_{b}\\ \frac{dw}{dt} & =\beta_{z}zV_{b}-d_{w}w\\ \frac{dP_{b}}{dt} & =b_{P_{b}}w-c_{2}P_{b} \end{array}\right. \end{array} \end{array}$$ (8)

where *z* represents the number of uninfected cells outside the liver and *w* the infected ones while *V*_*b*_ represents the number of HBV outside the liver in this extra-hepatic blood compartment, Ψ_*b*_. We omit the age-structure components for *V*_*b*_ in the Qesmi, et al. model here due to our focus on sinusoidal hepatic infection dynamics, but in turn add representation for the HBV protein p36 with an expression for *P*_*b*_ providing continuity for p36 diffusion from the liver to blood and back again. Note parameters are otherwise the same as in Qesmi, et al. (e.g., cellular birth & death rates, λ_*z*_ & *d*_*z*_, respectively) with alterations for our use such as the infected cell death rate notation (*d*_*w*_ here instead of their *a* but same value), and decay rates (*c*_2_) that are identical to ours used in the sinusoidal lumen (see Table 2). We refer the interested reader to [20] for further details including their parameter values and justifications for their use.

### Agent-based model

The deterministic formulations presented above naturally provide continuum solutions for mean levels of viral particles. This unfortunately does not capture intrinsic stochasticity such as probabilities of successful cytokine interference or cytolytic removal of infected hepatocytes. Moreover, the continuum methods do not address issues with observed variations in viral particle frequencies [43]. Thus, we deploy an agent based implementation of the above continuum HBV representations—directly following the work of Murray & Goyal [18].

The agent-based method entails drawing numbers from a Poisson distribution around what the deterministic model provides for the continuum averages. As the simulations progress from time *t*_*n*_ to *t*_*n*+1_, we distribute the solution data around the mean values from the deterministic model via the Poisson distribution as follows:

$$\begin{array}{c} \begin{array}{cc} V_{\Psi }^{n+1} & =V_{\Psi }^{n}+\sum_{i}P\;(\Delta t(J_{V_{\Psi }}^{+}\left(P_{\Phi ,i}^{n},R_{\Phi ,i}^{n}\right)-J_{V_{\Psi }}^{-}\left(V_{\Psi ,i}\right)-cV_{\Psi ,i}))\\ P_{\Psi ,i}^{n+1} & =P_{\Psi ,i}^{n}+P\;(\Delta t(J_{P_{\Psi }}^{+}\left(P_{\Phi ,i}^{n}\right)-cP_{\Psi ,i}^{n}))\\ V_{\Phi ,i}^{n+1} & =V_{\Psi ,i}^{n}+P\;(\Delta t(J_{V_{\Psi }}^{-}\left(V_{\Psi ,i}^{n}\right)-b_{r}e^{\left(-\lambda P_{\Phi ,i}^{n}\right)}V_{\Phi ,i}^{n}-\mu_{r}V_{\Phi ,i}^{n}))\\ C_{\Phi ,i}^{n+1} & =C_{\Phi ,i}^{n}+P\;(\Delta t(be^{\left(-\lambda P_{\Phi ,i}^{n}\right)}\left[R_{\Phi ,i}^{n}-V_{\Psi ,i}^{n+1}\right]-\mu C_{\Phi ,i}^{n}))\\ R_{\Phi ,i}^{n+1} & =R_{\Phi ,i}^{n}+P\;(\Delta t(bS_{\Phi ,i}^{n}-bR_{\Phi ,i}^{n}))\\ P_{\Phi ,i}^{n+1} & =P_{\Phi ,i}^{n}+P\;(\Delta t(a_{P}C_{\Phi ,i}^{n}-J_{P_{\Psi }}^{+}\left(P_{\Phi ,i}^{b}\right))). \end{array} \end{array}$$ (9)

Here, P(X) is the Poisson random number distributed about the mean *X* where we take the random number from the absolute value of *X* then multiply the output by the sign of *X*. Hence, the total number of sinusoidal HBV particles are summed over all hepatocytes. This gives the rather noisy data presented in the Results. Note, results presented are arithmetic averages of the number of runs computed (ranging from *n* = 5 to 20) unless noted otherwise. See Murray & Goyal for more details on this agent-based formulation, as we follow their implementation with minor variations for our spatial modeling framework.

### Parameters

Our multi-scale model is an assemblage of previously published models including a intracellular dynamic model [18] and an extra-hepatic blood model [20]. Their model parameter values are naturally utilised here in our assembly and all parameters displayed in Table 2 reflect published values. We briefly describe some of these parameters here. Conversion of *C*_Φ_ to *S*_Φ_ rate in parameter *a* = 50 *day*^−1^ is set to obtain levels of *S*_Φ_ and *R*_Φ_ at approximately 100-fold higher than *C*_Φ_. *P*_Φ*k*_, representing the largest of HBsAg proteins, are roughly 1000-times higher than HBV DNA levels; thus *a*_*p*_ = 1000*a*. Release of *R*_Φ_ and protein *P*_Φ*k*_ are set with a half-life of 1 day or *b* = *b*_*p*_ = log(2) *day*^−1^. *C*_Φ_, or cccDNA, half-life is estimated at around 50 days and hence *μ* = log(2)/50. Exported p36 is presumed to decay at the same rate as virions since they express the same envelope proteins, with both set at the rate *c* = 24 × log (2)/4 *day*^−1^. Steady-state values for the model aided in determining level of intracellular p36 or *P*_Φ*k*_; λ and its influence is set to 1/100,000 to obtain observed averages of 100,000 p36 particles per cell. Blood compartment parameters such as blood cell births (λ_*z*_), blood cell deaths (*d*_*z*_), and blood cell infectivity (*β*_*z*_), were taken from the Qesmi, et al. model [20] simulation suites aimed at finding viral loads permitting infection clearance. See source references for full descriptions of these parameters and their justifications.

Our focus here, however, is on the influence of spatial variations on persistence of infection. We thus perform our sensitivity analyses on the spatial distributions of key components including replication rates (e.g., *b*_*r*_, or conversion of *V*_Φ_ → *C*_Φ_), HBV invasion rates (e.g., *κ* or NTCP uptake), and immune cytolytic clearance (e.g., *δ*, or CYL). These spatial distributions are combined with different levels of immune response (e.g., *δ* or the adaptive CYL, and *u*, or the innate cytokine interference of HBV replication). We are unaware of any precise descriptions of such spatial distributions. Alternatively, observations of HBV replication apparently utilising similar transduction pathways to gluconeogenesis in fasting mice [28] combined with known elevations in gluconeogenesis near periportal regions [25] suggest a spatial element to HBV activity. We thus base our investigations on these and other inferred qualitative distributions observed in hepatic tissues; e.g., nutrient and oxygen levels higher near the periportal entry to the sinusoid. Other computational models of spatial impacts on liver activity, e.g., metabolisation of midazolam [27], arrange variant activity from peak to minimal levels in roughly linear fashion aligned with the periportal-pericentral axis as appropriate. Analogously, for this initial spatial investigation of HBV, we utilise straightforward linear gradients. Peak levels, for instance, of HBV replication are situated at the periportal end of the sinusoid coincident with elevated gluconeogenesis, and minimums at the pericentral for alignment with distributions of nutrients and oxygen available. The spatial distribution is illustrated in Results section ‘Gradient-based spatial heterogeneity’.

### Solution methods

We employ the following simplifying assumptions. The average volume of serum in human is 3L, and, based on reported numbers of hepatocytes in human livers ranging from 1.3 × 10^10^ to 3.61 × 10^11^ [44] [45], we use an average number of hepatocytes in a human liver of 6 × 10^10^ as in [18]. We also utilise a model of 5 parallel sinusoids of 30 hepatocytes each, resulting in a sampling of the total hepatocyte population at *N* = 150. This sampling of the hepatocyte population translates the initial inoculum of 2 × 10^9^ HBV DNA copies/mL (as utilised in [18]) into the scale of our model at an inoculum of 15,000 copies. This conversion from particle counts to concentration per *ml* of serum (as taken directly from Murray & Goyal) is given simply by: $V=\frac{N}{6x10^{10}}\times 3000\times \hat{V}$, for $\hat{V}$ the human equivalent of serum virions with presumed serum volume of 3 L, number of hepatocytes in liver estimated at 6*x*10^10^, and *N* the number of simulated hepatocytes in the model (as noted, for our implementation *N* = 150).

Above presentation of blood flow rates through the sinusoids indicate a sharply faster time scale than the HBV dynamics operating over days. Blood flow rates in the liver suggest utilising time steps on the scale of seconds. However, the HBV system inserts an intermediate *τ* of 30 minutes between this flow rate and the HBV dynamic evolving over days. Tractably managing the time-scale range compels our assuming the HBV dynamics at the seconds-scale are at steady-state. We thus compromise here by taking a time step of 1min, treating HBV distribution in sinusoids (outside hepatocytes) at time *t* as homogeneous. Pilot simulations at scale of seconds tend to validate this assumption, largely due to the rapid fluid flows through the relatively small sinus (scale 200 *μ*m), and we further observe homogeneous distributions of HBV particles after one simulated hour.

Numerical solution of the model equations were solved with customised solvers in MATLAB to maximise flexibility with an explicit first order, forward Euler scheme in time. As the time delay is fixed to *τ* = 30 min throughout, combined with a time step of Δ*t* = 1 min, we avoided interpolation issues with the delayed values through retention of 30 iteration histories. Comparison of pilot results with Matlab built-in solvers (e.g., dde23) and constant delays gave results comparable with our customised implementation.

The global process, for one iteration, of the whole model is the following scheme:

1. Update HBV dynamics in liver and blood compartment(s) solving Eqs (1, 2) & (8); Distribute particle numbers around mean value solutions according to Poisson distributions (Eq 9);
2. Apply CYL immune clearance, natural hepatocyte deaths & replenishment;
3. Solve advection / blood flow in blood sub-compartments to / from liver via Eq (7).

Stochasticity of the ABM model naturally requires multiple simulations and statistical analysis. We performed successively increasing numbers of simulations at the same parameter values and tested for convergence towards a mean and standard deviation—for the observed ‘Day of Clearance’ (DoC) when all HBV particles are removed from our simulated hepatocytes. At *n* = 5 simulations, mean levels for DoC at a variety of combinations for immune activity, *δ*, *u*, are stabilised. Standard deviations, are less well-behaved depending on level of immune activity. For instance, at *u* = 0.25 and *δ* at 0.6, standard deviations for DoC fall within a narrow range around +/- 20 days by 5 simulations and remain comparably so out to 30 simulations. Increasing *u* to 0.5, however, results in gradually rising standard deviations: +/- 5 days DoC at 5 simulations, +/- 10 days DoC at 10 simulations, and up to +/- 20 days for 20-30 simulations. We compromise here and typically perform *n* = 5 simulations presenting mean results presuming +/- 20 day standard deviations unless otherwise noted.

## Results

Our focus on spatial distributions within the sinusoids of the liver is inspired by known gradients of, for instance, oxygen and nutrients [25] that may in turn affect intracellular HBV dynamics, the uptake of HBV via the NTCP, or immune-cell responses. We investigated the following spatial-heterogeneities within the sinusoid (e.g., see Section ‘Gradient-based spatial heterogeneity within sinusoids’): (*i*) gradients of immune responses, particularly active immune cell cytolytic removal of infected hepatocytes; (*ii*) gradients of HBV replication efficiency; (*iii*) gradients of NTCP uptake of HBV and combinations thereof. Some consideration is made for differences between sinusoids themselves, e.g., variant liver lobule activity, as noted below. We establish the model first, however, with homogeneous distributions.

### HBV infection and clearance with a baseline model

**Fig 2:**
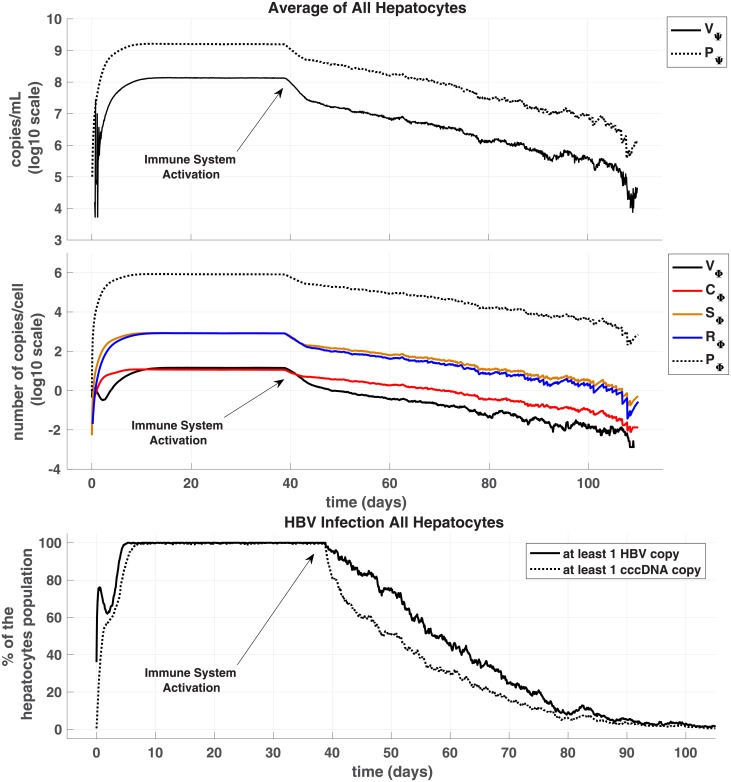
Evolution of hepatitis B infection homogenous case. With *n* = 5 simulations of agent-based stochastic model, we averaged results and present for sinusoidal viral particles (Panel A) and intracellular particles for hepatocytes (Panel B). Panel C plots percentage of infection across all hepatocytes where we define infected as at least one HBV or cccDNA copy. Results presented include extra-hepatic HBV replication and no cccDNA persistence in new replenishment hepatocytes. Note, scaling translates from the model values of, say, 7.5 HBV copies in our simulated liver to an entire liver at 10^6^ HBV copies/mL on the graph. Simulated inoculum of 2.10^9^ HBV copies/mL at t = 0, Δ*t* = *τ*/30 = 1min, *N* = 150 cells, *κ* = 0.3. Immune response activation at day 40 with CYL strength *δ* = 0.6 and Non-CYL strength *u* = 0.25. Note negative logarithmic values occur (Panels A and B) since the average number of copies/cell fall under 1, e.g., sums of HBV particles across all cells in our model translates into 1 particle per the population of 150 or log(1/150).

Initially, we consider the simplest case with identical distributions—or a homogenised liver—for comparison with our later heterogeneous depictions and include extra-hepatic replication. Fig 2 shows the evolution of the different particles involved in HBV infection dynamics from inoculum at day 0 to clearance day after activation of immune responses at day 40. Amount of particles in the sinusoidal space are rescaled to average human liver scale by multiplying our results by the ratio of cells number in the model over in average human (*N*/6^10^ = 2.5*e*^−9^ as we consider *N* = 5 × 30). Results throughout are all with immune clearances activated at 40 days or as noted by arrows throughout the figures. Activation at 40 days permits the immune-free system to settle to steady-state, and we can thus observe impact of immune activity without complications of transient behaviour.

Within 20 days the infection levels reach about 90-95% of steady state, but we allow simulations to run out to 40 days ensuring solutions are at roughly 99% of expected steady-state values. By the 40th day then, average number of *C*_Φ*k*_ copies in hepatocytes is 11.53, and each individual hepatocyte produced 819 complete and new virions (*R*_Φ*k*_) via the replication cycle (*C*_Φ*k*_ → *S*_Φ*k*_ → *R*_Φ*k*_). These numerical results for this spatially-homogeneous case compare well with our analysed equilibria of the DDE system (presented in S1 Appendix) with nominal deviations (relative error <1%).

Next, we varied either CYL or non-CYL immune responses independently or both in tandem. The CYL response strength, *δ*, was varied from 0 to as high as 1.2, and the non-CYL response strength *u* from 0 to 1. Higher CYL strengths are certainly possible in the model, but would require a non-physiological HT well above ranges observed of 0.7–1.0 [35] to maintain our constant liver cell population [18]. The particular case presented in Fig 2 with a CYL strength *δ* of 0.6 and a non-CYL strength *u* of 0.25 leads to acute clearance of HBV. We define the ‘day of clearance’ (DoC) as when there is no HBV particles, including no *C*_Φ*k*_, in the liver (hepatocytes & sinus), blood (outside the liver), or blood cells. We observe DoC here, on average, at 100 days post-inoculum, with a range from 92 to 115 days over the 5 simulations.

We note our simulations show that for CYL response weaker than *δ* = 0.6, infection leads to a chronic disease state unless non-CYL response compensates by elevation to *u* = 0.5 or higher. Alternatively, if the non-CYL response is weaker than *u* = 0.5, a chronic disease state results unless we again compensate by elevating CYL response (e.g., *δ* > 0.6, see data repository). Combinations of elevated CYL and non-CYL immune response levels greater than these leads to acute clearance of HBV with a speed naturally related to the strength of the immune system. Although the subtle interplay between the influence of *δ* and *u* provides a rich dynamic for exploration as in [18], we turn to focus on the impact of spatial distributions of the liver milieu on the HBV dynamic.

Results presented above were compared with the extra-hepatic HBV replication (in the blood compartment, Ψ_*b*_) inactivated. We compared DoC with and without this extra-hepatic replication using a Wilcoxon rank-sum test determining significant difference with *p* < 0.05. For a variety of combinations of immune activity, *p* values are all well over the 0.05 threshold: a minimum *p* emerges at about 0.54 (*δ* = 0.6, *u* = 0.25, *n* = 26). Although with *δ* and *u* at (0.3, 0.75), we do see one more successful clearance of infection without the extra-hepatic replication, the *p* value is nevertheless quite high (0.9, *n* = 22) suggesting influence of extra-hepatic replication is nominal. We further compared time to DoC at varied immunity levels with low rates of cccDNA survival to new hepatocytes (up to 2%) without observing statistical significance at *p* < 0.05 although with weaker CYL activity at *δ* = 0.6 significance almost emerges (minimum *p* = 0.053, *n* = 10, Wilcoxon rank-sum).

### Gradient-based spatial heterogeneity within sinusoids

We propose that, similar to known nutrient and oxygen distributions in sinusoids [25], the production, conversion and transport rates for HBV exhibit sinusoidal spatial distributions as well. The HBV dynamic may be affected in several ways including, as noted before, immune response efficiency, HBV replication cycle efficiency, or HBV hepatocyte invasion. For instance, the NTCP-bile-salt transporter (implicated as key to HBV uptake [11]) may exhibit higher activity at the periportal region, reflecting a higher concentration of periportal bile-salts, in turn providing more uptake ‘bandwidth’ for HBV invasion. Similar effects may be in force for other HBV aspects due to these known sinusoidal distributions, e.g., diminished replication activity at the pericentral region simply because of lower levels of nutrients available for protein manufacture.

**Fig 3:**
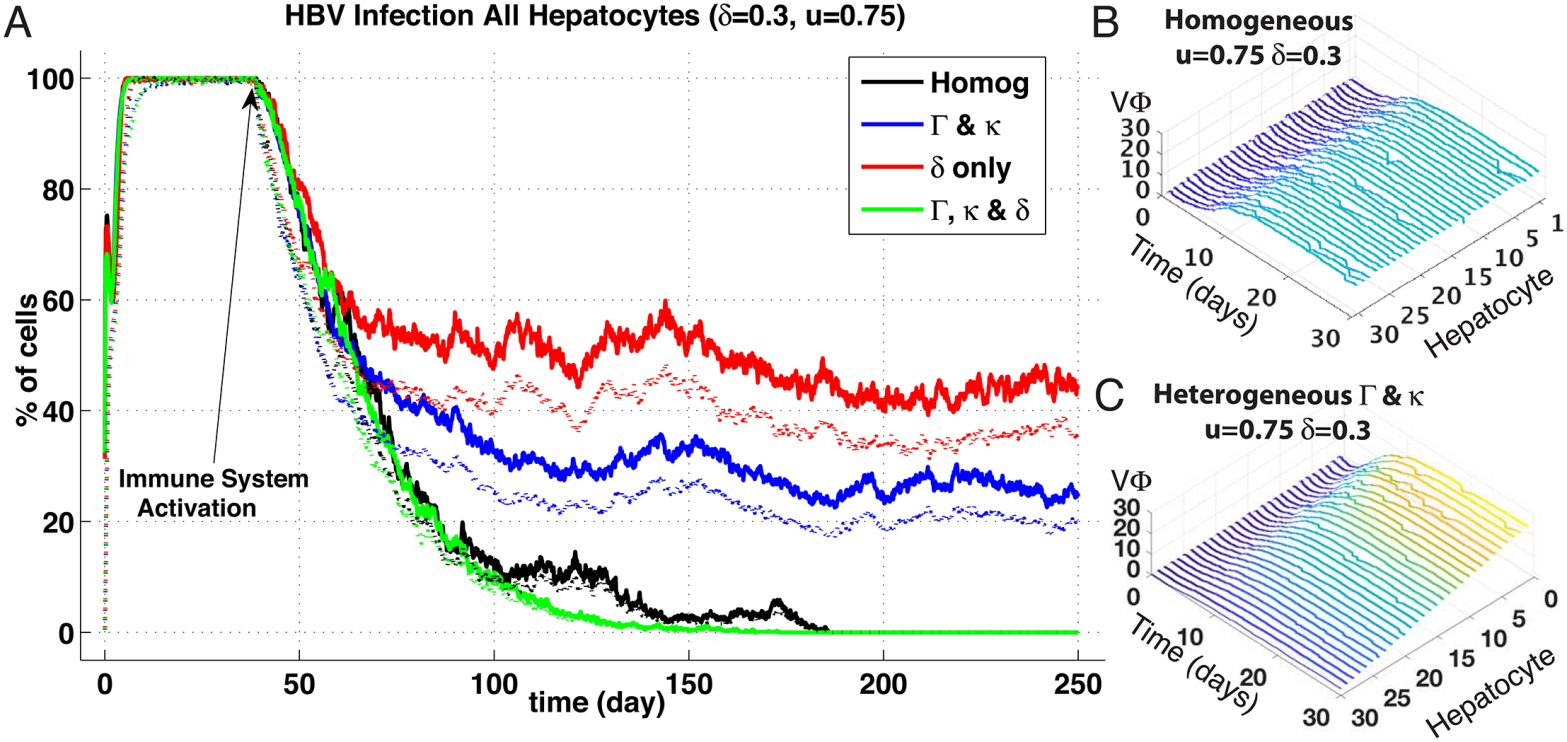
Spatial heterogeneities effects on HBV infection and clearance. (Panel A) Results are in percentage of cells; averaged over *n* = 5 simulations of agent-based stochastic model. Inoculum of 2.10^9^ HBV copies per mL at day 0 and immune responses activation at day 40 (*δ* = 0.3, *u* = 0.75). Legend: Solid lines represent percentage of cells in the liver with at least 1 HBV copy (*V*_Φ*k*_+*R*_Φ*k*_) and dotted lines percentage of cells in the liver with at least 1 cccDNA copy (*C*_Φ*k*_). cccDNA survival to replenishment hepatocytes was set to zero probability. (1) Homogeneous base line case; (2) Gradients of HBV replication cycle efficiency (parameters Γ and *κ*) (3) Gradients of *δ*, (4) Gradients of both (Γ, *κ*) and *δ*. (Panels B & C) Ribbon plots before immune activation (*t* = 1–30 days) showing intracellular viral particles (*V*_Φ_) for each hepatocyte (sinusoid 5). Hepatocyte number order reflects proximity to sinusoidal periportal entrance, i.e., #1 at entry point, and #30 at pericentral exit. Panel B shows particle counts over sinusoid with homogeneous spatial distribution of HBV replication rates (Γ) and uptake (*κ*) whereas Panel C shows distribution with linear gradient of same parameters. Peak parameter values situated at periportal hepatocyte #1 and minimal at pericentral #30. Note linear distribution of *V*_Φ_ corresponding to distribution of parameters with variance of 0.8; see section (3.2) for details.

We thus simulated these three proposed effects with comparisons to the homogeneous case, and meanwhile endeavoured to maintain mean parameter values identical to homogeneous model. Quantitative measurements of the degree of such spatial heterogeneities in the liver do not exist yet. Hence, we assumed a linear gradient with a variance of 80% around mean value of all the parameters involved in the HBV replication cycle (e.g., *a*_*min*_ = *a*_*mean*_ − 0.8 × *a*, *a*_*max*_ = *a*_*mean*_ + 0.8 × *a*) and distributed the parameters linearly over these minimal—maximal variations. Such a distribution is illustrated in Fig 3, Panels B and C, where the concentrations of intracellular viral particles across a sinusoid demonstrate this linear gradient effect. For convenience of reference, we define a vector encapsulating the entire suite of intracellular HBV replication parameters: Γ = (*a*, *a*_*p*_, *b*, *b*_*r*_, *b*_*p*_), and refer to this vector in subsequent variations of all the HBV replication rates; e.g, increases to *a*, *a*_*p*_, etc. This Γ is further varied in combinations with the NTPC uptake parameter, *κ*, as noted below.

The evolution of HBV infection after inoculum at day 0 and with immune response activation at day 40 (CYL response strength *δ* = 0.3, non-CYL response strength *u* = 0.75) for 3 following spatial distributions: a combination of varied HBV replication efficiency, Γ, and NTCP uptake, *κ*; a variation of CYL response, *δ*, alone; and a combined variation of (Γ, *k*) and *δ* (Fig 3A). cccDNA survival through to daughter or replenishment hepatocytes was set to zero probability. The homogeneous base-line case is included as well for comparison, yet note the immune response levels utilised here differ (reduced CYL and elevated non-CYL) than shown previously in Fig 2.

For the homogeneous base-line case (black-trace), the now weaker CYL combined with a stronger non-CYL results in DoC occurring at about the 180-day mark—significantly longer than the previously observed average of 100 days—perhaps reflecting the greater effectiveness of CYL action. A comparable result emerges with one of the spatially-distributed variants, where CYL gradients are combined with variations of the HBV replication parameter suite, Γ (green trace). DoC happens a bit faster (at around the 180 mark), but clearance nevertheless still occurs. By contrast, chronic infections arise in the other variants, at least out to end of simulated time of 250 days, where variations of HBV replication combined with uptake (blue trace) reduce the level of chronic infection by about half compared with variations of CYL alone (red trace).

**Fig 4:**
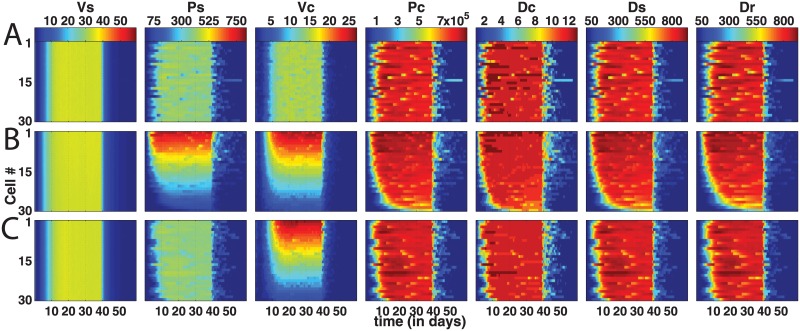
Spatial plots showing viral particles levels over cells within a single sinusoid per row (‘unit model’ #1) in three different parameter sets. Plots are averages of *n* = 5 simulations that are evolution of only one sinusoid for 3 cases of agent-based stochastic model: (a) Homogeneous baseline case, (b) linear gradient-based heterogeneities of Γ and *κ* combined, and (c) linear gradient-based heterogeneity of *κ* alone. Columns correspond to the suite of either sinus particles (*V*_*s*_, *P*_Ψ_, two leftmost columns) or the full cohort of intracellular particles (*V*_Φ*k*_ to *R*_Φ*k*_, 3^rd^ column to last). For each plot, x-axis represents the time in days, y-axis represents spatial location so that *y* = 1 is the first hepatocyte cell at the periportal entry of the sinusoid and *y* = 30 is the 30^th^ hepatocyte cell at the pericentral end of the sinusoid. Colour bars above each plot show levels of each particle for each respective column across the rows. Inoculation at day *t* = 0 and activation of immune responses (CYL and non-CYL) at day *t* = 40 (*δ* = 0.9, *u* = 0.25) similar to prior results shown, and cccDNA survival probability to daughter hepatocytes set to zero again. Variance for gradient heterogeneities: 0.8. (See Fig 3 for depiction of these gradient-based distributions.)

The averaged transient plots over all sinusoids obscures the spatial detail of HBV dynamics, and we present samples of our entire sinusoid suite displaying HBV particle counts for each hepatocyte in the first of our five simulated sinusoids, or ‘Sinusoid #1’ (Fig 4). Presented are three spatial distribution variations: (a) all homogenous parameters, (b) heterogeneous Γ & *κ*, and (c) heterogeneous *κ* alone. Immune response levels are with CYL activity significantly stronger than shown previously (*δ* = 0.9) coupled with a relatively low non-CYL HBV replication interference (*u* = 0.25), again activated at *t* = 40 days.

The influence of spatial heterogeneities clearly emerges, where we observe viral levels requiring longer to comparably infect the entire sinusoid. With NTCP uptake levels diminished (reduced *κ*) at the pericentral region (hepatocyte #30) combined with HBV replication inefficiencies (reduced Γ overall), the virus is not fully entrenched as in the homogeneous case when CYL activates at 40 days. With NTCP uptake alone subject to spatial gradients, infection of HBV displays a less-distinct drop off towards the pericentral region, and *C*_Φ*k*_ requires about half as may days to colonise the pericentral hepatocytes as when the HBV replication levels are also reduced (compare rows (b) & (c)). In fact, it is primarily *V*_Φ*k*_ that demonstrates the most vivid contrast with the homogeneous results (although *P*_Ψ_ does as well with impeded levels of Γ), where extreme levels at the sinusoid ends are either higher (periportal of 25 *V*_Φ*k*_) or lower (pericentral of 0 *V*_Φ*k*_) than the homogeneous with around 15 *V*_Φ*k*_ throughout.

An interesting outlier occurred for the homogeneous results—note the apparent resurgence of infection for hepatocyte #15 indicated by the white line appearing around day 45 for all viral particles except complete virions, *V* (Fig 4, row (a)). Recall, these are averaged results; in actuality, only one of the suite of simulations presented an infected hepatocyte surviving CYL clearance. This lonely infected hepatocyte, although continuing to produce HBV particles well after CYL initiation, nevertheless failed to infect any other hepatocytes, either in this sinusoid #1 or any other, and was eventually eliminated by the immune clearance system within the 60 days of simulated time. The result of a surviving infected hepatocyte despite a strong CYL response (recall, *δ* = 0.9) here is likely due to the probabilistic process utilised in our modeling scheme. We attempted to reproduce this result but due to stochasticity of the model (and time constraints) did not observe a repeat occurrence. However, we did perform simulations with one hepatocyte infected with one copy of *C*_Φ*k*_, and contingent on CYL activity, observed total reinfection of the liver population (see Discussion).

The third row represents the isolated impact of NTCP variants where only the NTCP uptake rate along the sinusoid is heterogeneously distributed. Now the entire HBV cycle is affected and shows a gradient distribution in the HBV particle levels. Notably, if we altered the variance around the mean levels of NTCP uptake, *κ*_*mean*_, we observe quite similar distributions. For instance, with variances at 0.4 (instead of 0.8), spatial distributions still follow the linear gradients of *κ*, but with levels that naturally reflect the peak and minimal uptake rates (see data repository). Alternatively, if we increase the variance, we can obtain plots similar in appearance to those in the second row (Fig 4b).

Similar to the homogeneous case, disabling extra-hepatic HBV replication results once more in a nominal impact on DoC with spatial distributions of key parameters. At the same levels of immune activity with extra-hepatic replication, distributing uptake, replication and cytolytic clearance gives the most noticeable effect on clearance—yet remains statistically insignificant (Wilcoxon rank-sum, *p* = 0.42, *n* = 22). Overall we do not observe a significant effect on clearance with extra-hepatic replication with either homogeneous or heterogeneous spatial distributions of Γ, *κ*, or *δ*.

With enabling cccDNA survival through to replenished hepatocytes at a 2% probability, the heterogeneous distributions have an observable effect significantly reducing the number of simulations achieving DoC but only with lower non-CYL activity. At *u* = 0.25 and *δ* of 0.6, the number of simulations reaching DoC by 250 days is reduced by 70%. Alternatively, at all other immune activity levels—with homogeneous spatial distributions—we do not see any statistically significant impact of cccDNA survival at our estimated level of 2% for the simulated time-frame.

### Organ-scale spatial heterogeneity

Liver heterogeneities likely extends beyond the micro-scaled sinusoids, and we thus consider distributions of HBV replication and / or CYL immune activity over the different representative sinusoids in our liver model. We limit ourselves to investigating organ-scale heterogeneities with extra-hepatic replication activated in compartment Ψ_*b*_ and no cccDNA survival to replenishment hepatocytes. With one sinusoid in one liver ‘lobular’ region (e.g., sinusoid #1) exhibiting increased immune clearance (CYL, *δ*), this particular sinusoid and presumably its surrounding liver tissue results in faster clearance without showing any impact on the *overall* clearance for all sinusoids. We can compensate for this faster localised ‘lobular’ clearance by increasing rates of HBV replication, Γ. The net difference with base-line cases is higher probability of liver damage due to elevated HBV replication and CYL response. Alternatively, with an elevated Γ in the lobule represented by sinusoid #1, but without an increased challenge of immune clearance (e.g., base-line *δ*), we observe a global effect on the whole liver. Despite successful immune activity in other sinusoids, the spatial heterogeneity of elevated Γ in one sinusoid leads to a chronic infection (see data repository).

By contrast, a decreased immune clearance (lower *δ*) in sinusoid #1 requires longer for the liver system to reach DoC—depending on the degree of clearance impedance. For instance, halving *δ* leads to longer infections before we observe DoC at roughly 25% longer compared with base-line (homogeneous case, see data repository). We can essentially determine the DoC through the a single sinusoid’s CYL effective activity: reducing immune responses more may take 30–50% longer, and persistent infections result, for the simulated time scale of 250 days, if *δ* is reduced an order of magnitude. Interestingly, for a relatively modest immune activity level (*δ* = 0.6, *u* = 0.25), we can obtain DoC around 100 days for the homogeneous case, but not the heterogeneous with sinusoid #1 exhibiting slightly reduced CYL activity (*δ* = 0.6 in the 4 normal sinusoids and *δ* = 0.3 in the weaker sinusoid).

### The NTCP

As noted, the NTCP is key to HBV invasion [11], and we thus explored impact of inhibiting its behaviour on our model liver. We applied a modifier, *u*_2_, to the NTCP flux term thus,

$$J_{V_{\Psi }}^{-}\left(V_{s}\right)=\kappa \times \left(1-u_{2}\right)V_{s}\left(t-\tau \right),$$ (10)

permitting adjustment of NTCP activity without directly changing the uptake parameter, *κ*, in essence representing action of a pharmacological antagonist. Initially, we applied *u*_2_ homogeneously at varying levels over [0.25, 0.5] with a Δ*u*_2_ of 0.05. Combined with an immune activity of *δ* = 0.3 and *u* = 0.5, simulations fail to present any DoC within 250 days—without any cccDNA survival to replenishment hepatocytes. Although there is certainly a reduction in the percentage of infected cells (e.g., ∼95% down to ∼80% infected, for *u*_2_ = 0, 0.25, respectively), DoC results only if NTCP uptake is blocked by at least 50% (see data respository).

**Fig 5:**
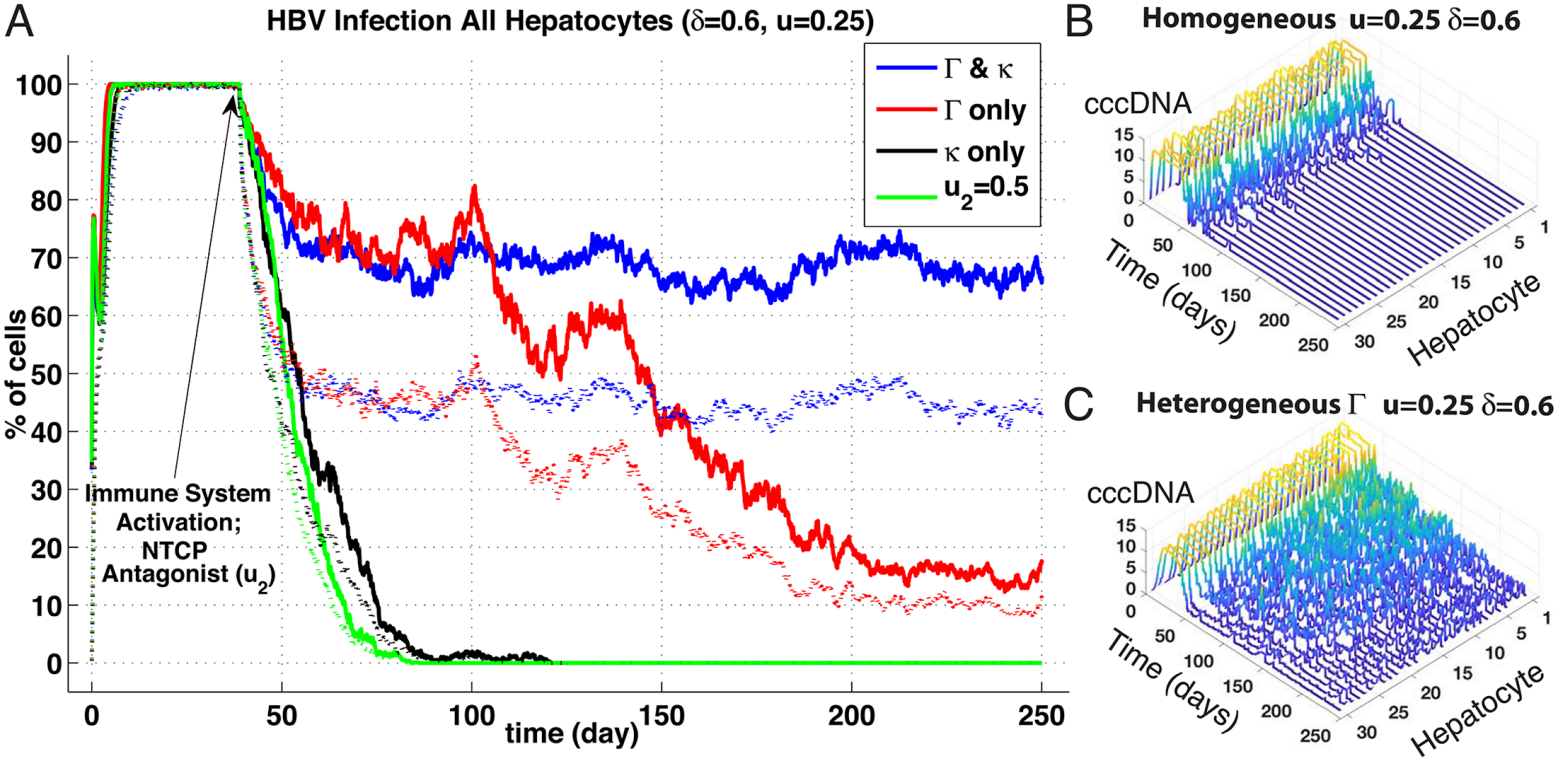
NTCP spatial heterogeneities effects on HBV infection and DoC. (Panel A) Results plotted are in percentage of cells; averaged over *n* = 5 simulations of agent-based stochastic model. Inoculum of 2.10^9^ HBV copies per mL at day 0 and immune responses activation at day 40 (*δ* = 0.6, *u* = 0.25) Plain lines represent percentage of cells in the liver with at least 1 HBV copy, Dotted lines represent percentage of cells in the liver with at least 1 cccDNA copy. cccDNA survival probability to replenishment hepatocyes set to zero. (blue trace) Gradient-based heterogeneities of HBV replication cycle efficiency, (red trace) Gradient-based heterogeneities of HBV replication cycle efficiency excluding the HBV uptake from sinusoid to cell (parameters Γ), (black trace) Gradient-based heterogeneity of the HBV uptake (parameter *κ* linked to NTCP presence), (green trace) Homogeneous base line case with activation of NTCP blocking (homogenous strength *u*_2_ = 0.5) at day 40. Heterogeneities linear distributions with variances set to 0.8 (see text). (Panels B & C) Ribbon plots showing progression for cccDNA counts over all 30 hepatocytes (sinusoid 5) with spatially homogeneous (Panel B) and heterogeneous Γ (Panel C) up to *t* = 250 days. Note, homogeneous distribution at these immune activity levels leads to clearance at around 100 days.

We next explored impact of heterogeneously active NTCP in solo as well as in combination with other components, such as HBV replication activity via Γ. Fig 5 compares 4 different parameter distributions at an immune response strength with higher CYL activity than shown previously (*δ* = 0.6, *u* = 0.25) with heterogeneous Γ where we do not obtain a DoC—regardless of the NTCP uptake activity. Indeed, if *κ* is spatially varied in concert with Γ, infection levels at end of simulated 250 days is substantially higher than with homogeneously active NTCP (roughly 70% vs. 20%, compare blue and red traces). Notably, if we enable cccDNA survival to replenishment hepatocytes at a level of 2%, simulations naturally do not achieve any DoC within 250 days, but infection levels are significantly higher and comparable to results with both Γ and *κ* in coincident spatially distributions.

Achieving DoC within our simulated 250 days requires either an antagonist homogeneously blocking NTCP at 50% (green trace), spatially-distributing NTCP uptake alone (black trace), or no spatial variations at all (ribbon plot, Panel B). Note, disrupting NTCP uptake—either uniformly over the sinusoid via *u* or a spatial distribution—initially reduces viral particle levels faster than without disruption, although DoC may be delayed somewhat (on average of 5 days, heterogeneous *κ*). Perhaps unsurprisingly, variation of spatial distributions for NTCP with and without extra-hepatic replication has no significant effect, and in fact are nearly identical distributions (*p* = 0.91, *n* = 25), similarly to our previous comparisons with Ψ_*b*_ activated or not. We do observe, naturally, reduced infection levels where *κ* is lower at the pericentral hepatocytes, permitting immune clearance an easier task in that area, but alternatively higher NTCP uptake levels on the other sinusoidal end that lead to faster reinfection. The balance of these effects is evidently a slightly faster time to DoC than observed with a spatially homogeneous NTCP uptake. Nevertheless, spatially variation of the NTCP uptake levels across the sinusoid does not prevent clearance unlike when combined with gradients of HBV activity—without resorting to any pharmacological and homogeneous inhibition through the parameter, *u*_2_.

## Discussion

We have assembled published mathematical models of intracellular HBV dynamics, sinusoidal-hepatocyte interactions and blood-compartment HBV infection into a single and modular multi-scaled representation of a whole-organ liver HBV model. Our aim was to explore the impact of spatially distributed components involved in HBV activity that may be distinctively relevant to characterising HBV colonisation given the intrinsic spatial complexity of the hepatic environment. With some modifications to the sub-model components we fortunately inherited [27] [18] [20], we studied such spatial impacts through gradient variations of key parameters. These included intracellular replication of HBV (parameter Γ), the activity of immune responses including the CYL or elimination of infected hepatocytes (parameter *δ*) and the non-CYL such as interdiction of HBV replication (parameter *u*), and of course the variation of HBV uptake through the NTCP transporter (parameter *κ*).

Spatially-graded parameters such as the immune system response levels are inferred possible in the sinusoids given densities of, say, hepatic macrophages such as Kupffer cells exhibiting heavier weighting around the portal triad inlets [26]. These spatial aspects extend as well to the simple levels of nutrients and oxygen or gluconeogenesis signaling pathway distributions through the sinusoid [25], that may in turn impact HBV replication dynamics due to restrictions on available components for protein manufacture. Spatially-organised calcium (*Ca*^2+^) waves from central venuels to the portal regions, responding to hormones such as vasopressin, involve coordination of hepatic activity by localised sinusoidal ‘sensor’ cells evidently expressing higher levels of receptors [46] [47]. Such distinct spatial configurations suggested the distribution of NTCP uptake may also be subject to spatial gradients, although typical expressions appear to be evenly distributed [21]. Unfortunately, precise experimental details of any quantitative distributions for these spatial aspects is not available. We are unaware of any observations of spatially-localised HBV expression other than suggested by observed coincident utilisation of signal transduction pathways for HBV replication and gluconeogenesis [28] [25]. Moreover, there may be further structural influences on replication or immune activity and transport via blood or inter-cell transmission of HBV that likely complicates this picture. We focus though our initial investigation of spatial distribution impacts to the above noted heterogeneities within the sinusoids with some attention to heterogeneities over the whole organ as well.

### Distributions of immune cell activity

Little surprise occurs with spatially-confined immune activity. CYL distributed over the sinusoid at a variance of 0.8 from the mean does not exhibit a DoC unless *δ* or *u* are pushed substantially higher than what is necessary in the homogeneous case. For instance, with *δ* increased to 0.6 with *u* at 0.75 we obtain DoC at around 100 days, or *δ* at 0.9 with *u* at 0.25 at around 150 days, yet with homogenous *δ*, DoC results in roughly 60–80 days at the same immunity levels (see data repository). Alternatively, HBV dynamics and NTCP uptake elevated at the periportal region tends to overwhelm homogeneous *δ* and *u* if they are too low to compensate. Interestingly, with a distribution of Γ and *κ* over the sinusoid, we see a higher sensitivity of DoC to elevated *u* responses. If *u* is set at our highest level of 0.75, the CYL activity may be kept much lower (*δ*_*mean*_ = 0.3) and obtain clearance well before a homogeneous CYL distribution at the same *δ* removes HBV from our simulated liver (see data repository).

Moreover, it appears that the heterogeneous distributions for HBV dynamics and uptake give higher peak infection levels for *V*_Φ*k*_ and *P*_Ψ_, but not the other particles. This is likely due to simply higher concentrations of HBV corresponding to peaks of Γ and *κ*. In turn, we see these heterogeneities impacting times to clear by varying degrees depending on whether immune responses are similarly distributed. If CYL is evenly distributed, DoC is typically delayed by around 15% when combined with gradients of Γ and *κ*, and this delay is reduced to a modest 5% if the CYL *δ* parameter is similarly graded across the sinus. However, if *δ* is spatially distributed alone where infection and HBV replication is equivalent over the hepatocytes in the sinusoid, the CYL activity struggles to reach clearance, resulting in substantial delays of roughly 40%, compared with similar parameters homogeneously distributed (see data repository).

### Combinations of distributions

Hence, clearance appears quite sensitive to combinations of spatial distributions. Consider, these distributions of parameters are +/- variance about the mean, so, for instance, we obtain NTCP uptake rates higher than the mean at the periportal end. When spatially distributing *κ* in tandem with Γ in such a manner, more HBV uptake occurs at precisely the same regions where HBV replication is higher—an unfortunate coordination of elevated activity leading to more effective cycling of HBV replication, release and uptake. Alternatively, the infection levels fall over time when Γ is combined with a homogeneous—and average—NTCP level across the sinusoids, since uptake of virions at the higher replication sites is lower.

We can obtain DoC within our simulated 250 days by combining these concentrated distributions of Γ and *κ* with distributions of CYL activity across the sinusoid as well. Concentrating CYL action at the periportal entrance appears to counter the unfortunate and combined impact of elevated HBV replication with increased invasion. Although we did not spatially vary non-CYL activity via *u*, this nevertheless suggests that the hepatic distribution of immune cells may reflect localisations aimed at more effectively disrupting viral invasions. This may particularly be the case if the uptake of HBV is further spatially configured with higher activity at the periportal region, e.g., NTCP expression levels elevated where HBV initially enters the sinusoid.

### NTCP distributions

Our results suggest that simply varying the NTCP activity alone actually accelerates clearance of HBV when compared to a purely homogeneous distribution (Fig 5). Although impedance of NTCP uptake with no spatial gradient applied to *κ* clears the infection substantially faster, merely distributing *κ* along the sinusoid from a maximum of 80% over the mean to 80% below results in DoC within a comparable time frame. We are, however, unaware of any experimental observation of spatially-distributed NTCP activity within the sinus. We suggest then, due to our *in silico* results, that a spatially-distributed expression of NTCP may be related to establishment of chronic HBV—in tandem with spatial distributions of HBV activity, or Γ. That is, individuals with heterogeneous expression of NTCP are more vulnerable to persistent HBV infection if the replication activity of HBV is similarly distributed as indicated by persistent infections shown in both instances of Figs 3 and 5 for the combined Γ & *κ* traces. This requires further investigation though from either experimental confirmation of NTCP distributions or continued *in silico* testing of more varied spatial configurations.

### HBV replication distribution

The known spatial distributions of nutrients and oxygen over the length of a sinusoid from the periportal to pericentral may have implications for HBV replication rates—as well as observations of HBV utilising similar transduction pathways as gluconeogenesis [28]. Simply, more nutrients concentrated at the periportal zone may result in higher levels of HBV production—a primary inspiration for this spatial HBV study. Given adequate levels of immune activity, elevating HBV replication rates via Γ at the periportal region appears to have only nominal effect on DoC unless we concentrate uptake levels through the NTCP (*κ*) as well (see Fig 3). However, we see instances of failure to clear infection by 250 days with a rather strong CYL activity by merely concentrating these HBV replication levels at the periportal end that otherwise results in clearance if homogeneously distributed (Fig 5). Alternatively, a slight elevation of cytokine activity eliminates this effect; if we increase *u* to 0.5, we do not observe persistent infection (DoC around 80 days, see data repository). Hence, our results further suggest that if indeed HBV replication is distributed in the sinusoid similarly to nutrient and oxygen levels or gluconeogenesis—along with suitably weak levels of immune responses—persistent infection results.

### Extra-hepatic blood replication

We included the blood-compartment HBV replication model from Qesmi, et al. [20] to observe impact of such a reservoir on overall clearance within our simulated liver. Somewhat surprisingly, we observed little or no significant effect on days to clear infection. All our simulations with and without HBV production in Ψ_*b*_ resulted in no significant difference over a wide variety of immune level activities as well as spatial heterogeneities within sinusoids. This does not exclude the possibility of reinfection, however, from a solo extra-hepatic HBV cccDNA, for instance, as suggested by reinfection of transplanted livers. We thus also considered instances where a single cccDNA remains in an infected host either within the liver or without.

### cccDNA survival

Taking account for a low probability of 2% cccDNA survival by representing a proportion of new hepatocytes as carrying over the infection to daughter cells appeared to have a statistically significant effect only when spatial distributions of HBV replication, uptake or immune activity is applied as well. With homogeneous spatial distributions of all aspects, although we observe nearly significant impacts, but not quite at *p* levels of 0.05. We did vary the level of cccDNA survival probability above our 2% figure, and of course at higher levels significance results. The exact amount of survival may in fact be even less than 2% but this is uncertain. Nevertheless, combining a low level of cccDNA survival probability with homogeneous spatial distributions is not significant to persistence of infection hat may have implications for modeling efforts excluding any spatial considerations. By contrast, we see spatially-heterogeneous distributions of key aspects (replication, uptake, etc.) as sensitive to even such low survival probabilities, requiring longer times or stronger immune responses to DoC.

### Infection resurgence

**Fig 6:**
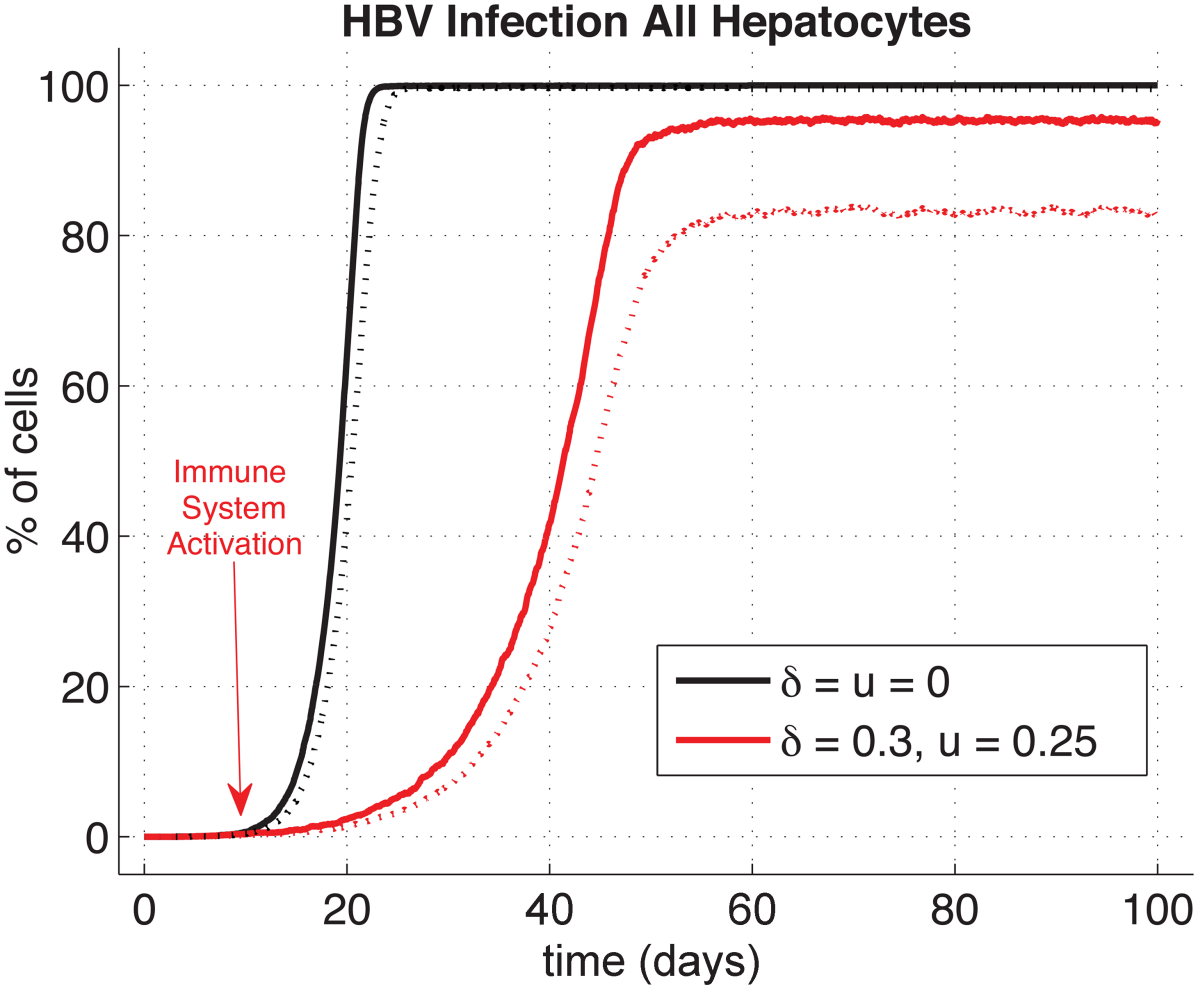
Reinfection of transected or transplanted liver with solo cccDNA copy. Results here with increased hepatocyte population than in other results: 50 sinusoids of 300 hepatocytes or 15,000 total cells, all homogeneously distributed (no spatial gradients of any parameters). This corresponds to 0.006% of cell population with 1 cccDNA. cccDNA survival probability to replenishment hepatocytes here set to zero. (1) Evolution of HBV from only 0.006% cells infected with 1 cccDNA without any immune response (2)(1) Evolution of HBV from only 0.006% cells infected with 1 cccDNA with activation of a weak immune response at day 10 (*δ* = 0.3, *u* = 0.25) Legend: Plain lines represent percentage of cells in the liver with at least 1 HBV copy (*V*_Φ*k*_+*R*_Φ*k*_). Dotted lines represent percentage of cells in the liver with at least 1 cccDNA copy.

Our encounter with a single surviving cccDNA in one of our simulations with zero probability of cccDNA persisting through to replenishment hepatocytes (see Fig 4a) compelled consideration of a scenario with a single cccDNA copy either residing in a lone hepatocyte or an extra-hepatic cell. Such an unfortunate circumstance may arise during transection or transplantation of an infected liver, and we observed in our simulations rather dramatic reinfections of nearly the entire liver population (Fig 6). The rapid and thorough reinfection observed here, however, is contingent on immuno-suppression for a transplantation subject. Typically, immune activity must be suppressed for such procedures and we represented these with either a rather weak immune response (*δ* = 0.3 & *u* = 0.25) or none at all. Persistent infection then results as we observed earlier with homogeneous parameter distributions, unless we increase the immune response accordingly; e.g., DoC again emerges if, say, *u* is increased to 0.75 as shown previously (Fig 3). Otherwise, the majority of our simulations do not result in reinfection since the lone infected cell typically does not survive long enough to produce any viable infective virions.

Similarly, variations of HBV replication rates or immune activity over the entire liver may establish infective reservoirs of HBV. For instance, if a sinusoidal region suffers from obstructions that impede immune clearance of infected hepatocytes (via, say defenestration of the sinus epithelia [7]), the liver will experience continuous reinfections from that region. Alternatively, if HBV is less efficient in a sinusoid perhaps due to higher interferon concentrations disrupting viral replication, we observed simulations with somewhat more rapid times to DoC, as expected (see data repository).

The impact on HBV dynamics due to combinations of such variations over the lobular regions on the scale of the whole organ are difficult to readily characterise, particularly over longer time scales than considered here where cytolytic damage and collateral effects such as cirrhosis and fibrosis introduce substantially more complications to the already complex spatial environment of the liver. Nevertheless, our relatively simple exploration of these spatial considerations strongly suggests that a single malfunction of the immune response in a restricted region of the liver may be all the opportunity HBV requires to reinfect an otherwise healthy organ.

### Implications for treatment

The extraordinary resilience of a single HBV cccDNA leading to resurgent infection in our simulations is consistent with clinical observations [3]. A straightforward suggestion for treatment from our results is organ-wide pharmacological blockage of NTCP uptake at a sufficient level. However, bile-salt transport is the primary function of the NTCP commandeered by HBV for hepatocyte invasion; disruption of bile-salt transport and processing likely leads to myriad pathologies in and of itself [21]. Of course, if the level of blockage is appropriately calibrated, successful disruption of HBV and maintenance of liver processing of bile-salts may be possible, but, modulation of NTCP activity in concert with immune suppression complicates this approach. For instance, innate immune release of interleukin-6 that interferes with HBV replication and downregulates NTCP also blocks apoptotic mechanisms and may even assist HBV progress [12]. We further observed here rather weak immune activity levels require an increase in NTCP blockage for clearance (*δ* = 0.3, *u* = 0.25 with *u*_2_ = 0.75, see data repository) that again may lead to unintended and self-defeating collateral disruption of liver function.

Alternatively, amplifying CYL removal of infected hepatocytes (by unknown means) may further trigger the familiar sequence of tissue damage and eventual carcinoma [4], and is sensitive to HT rates. Perhaps elevation of CYL rates in combination with increased cellular regeneration by virtue of manipulating the influence of *Ca*^2+^ on cellular replication (among other mechanisms [46]) may address this issue, but we are just speculating. Our results do indicate a higher effectiveness of CYL over non-CYL for obtaining clearance, e.g., *u* levels typically must be raised significantly higher to obtain DoC as opposed to necessary elevations of *δ*, but also provide a less-damaging mechanism for containment of established HBV.

Our results showing sensitivity of persistent infections to spatial gradients of HBV replication and particularly in combination with NTCP uptake unfortunately does not suggest a treatment *per se*, but rather an explanation. Chronic HBV persistence occurring in roughly 5% of infected individuals may be due to a smaller proportion of individuals expressing NTCP heterogeneously throughout sinusoids in tandem with similar distributions of HBV replication perhaps due to known gradients of oxygen and nutrients. Furthermore, distributions of immune activity with, say, elevated levels at the periportal entrance of the sinusoid coincident with the peak levels of NTCP and Γ may compensate as our results suggest where we observe clearance (e.g., Fig 3 green trace). HBV may alternatively establish a foothold in the periportal hepatocytes if immune activity distributions are not concentrated at the same site of elevated NTCP uptake and HBV replication. However, this scenario of course is subject to whether the NTCP and HBV replications levels or CYL distributions for that matter indeed exist in this fashion. Alternate spatial configurations may complicate this picture, and our investigation here was moreover rather limited in scope of distributions explored.

### Limitations

The above discussion reinforces the point: we do not know if NTCP are expressed or active in a spatial distribution through the sinusoids, or, for that matter, the HBV activity itself. If indeed they are, then our results suggest such distributions are important particularly if in tandem. Such variations are not unlike the numerous other mechanisms expressed with spatial distributions in the liver. Regardless, we restricted our exploration of such spatial distributions for NTCP and other parameters (e.g., Γ, *δ*, *κ*, etc.) to rather simple linear gradients around a mean, and typically with identical orientations (e.g., maximal-minimal from periportal-pericentral). Physical distributions likely follow different patterns and may be in reverse orientation to those investigated here such as reported with vasopressin receptors [46].

Responses of immune activity are fairly idealised here as well. Cytokine release in the model is essentially instantaneous and distributed throughout all sinusoids, whereas diffusion through tissues are of course occurring upon HBV challenge. Additionally, we include no action of any viral-inhibiting antibodies that may substantially influence the HBV activity, perhaps further modifying any spatial effects we observed. We did distribute CYL effects in some cases, but did not account for localised activity of innate immune responses or the likely influence of the innate on the adaptive—not to mention the apparent manipulation of the immune cells by HBV itself [13].

Replenishment of hepatocytes is further instantaneous in our model: there is no delay for recovery of eliminated infected cells. Also, we do not represent any tissue damage impact; e.g., defenestration of epithelia from cytolytic activity that in turn disrupts effectiveness of immune surveillance for infected hepatocyte detection. This in turn reveals the rather limited time scales we explored of up to 8 months that is enough to determine whether an infection may be established, but not whether, say, a latent infection may resurge due to longer-term effects.

Meanwhile, there is no HBV dynamic in the sinus other than transport of complete viral particles *V*_*s*_ and associated protein. If indeed HBV may infect red blood cells (or the venous epithelia), then yet another reservoir of HBV is available for reinfection, in addition to the observed occurrence of HBV in the pancreas, kidney and skin [48]. These suggest future investigations may need to account for such reservoirs and the likely variations of HBV dynamics in each cell type.

Moroever, the sinusoidal blood flow velocity we calculated at 183 *um*/*s* may be considerably faster than actual rates in the sinus, and may be linked to relatively rapid spread of infection in our spatial model compared with some work in chimpanzees [49]. Subsequent modeling efforts we intend with inclusion of more complex vasculature and fluid flow representations [40] will naturally widen our current focus of spatial distributions.

## Conclusion

The favoured target for infection by HBV, the liver, provides a rich and complex environment replete with spatial reticulations for the organ to perform myriad functions crucial to survival. Our mathematical model aimed at exploring the impact of a small subset of these features indicate HBV may be exploiting this intrinsic spatial complexity of the liver. Spatial configurations of key components such as NTCP transporter uptake of HBV in concert with similar distributions of intracellular HBV replication leads to chronic infections over our simulated time scales. Unless immune responses are suitably and coincidentally distributed, HBV will persist in an infected liver according to our results here. This result hinges though on experimental confirmation of spatial gradients of NTCP expression, HBV activity or immune clearances in the sinusoids. If such gradients do exist, then, manipulating the immune responses in chronically infected individuals to achieve such a coincidental and targeted distribution would clear the infection, yet this is unlikely. Hence, as our results and others suggest, suitably calibrated disruption of NTCP uptake appears most straightforward and promising for treatment. Regardless, our model indicates establishment of chronic HBV is related to spatial distributions of relevant components in the liver sinusoid. We thus encourage and await future experimental investigations of the HBV dynamic including consideration of these complicating yet apparently influential spatial features.

## Supporting information

S1 Appendix (PDF)
