# Supplementary material for: A multi-scale spatial model of hepatitis-B viral dynamics
Source: PLoS One. 2017 Dec 7;12(12):e0188209. doi: 10.1371/journal.pone.0188209 (PMC5720747; doi:10.1371/journal.pone.0188209)
Supplement: S1 Appendix — (PDF) [file pone.0188209.s001.pdf]

# A MULTI-SCALE SPATIAL MODEL of HEPATITIS-B VIRAL DYNAMICS

Quentin Cangelosi[1] Shawn A. Means[2*] Harvey Ho[2]

**1** Computational and Mathematical Engineering, Institut National des Sciences Appliquées, Toulouse, France
**2** Auckland Bioengineering Institute, University of Auckland, Auckland, New Zealand

* s.means@auckland.ac.nz

## Supporting Information

**S1 Appendix. Steady State Analysis**
An analytic study of the DDE system (Equations (1) and (2)) can give an overview of the HBV particles evolution. As this is the main part of the model we can expect a similar overall evolution of the entire model. Assuming a steady state exists (as suggested by simulations), all time derivatives must, by definition, be equal to zero once the steady state reached: $\frac{\partial \bullet}{\partial t} = 0$. Thus, all variables have constant values: $V_{\Phi k}(t) = V_{\Phi k}(t-\tau) = V_{\Phi k}(t+\Delta t)$. In that way, the DDE system become:

$$\begin{cases} b(1-e^{-\lambda P_{\Phi k}})R_{\Phi k} - cV_\Psi - \kappa V_\Psi = 0 \\ b_p P_{\Phi k} - cP_\Psi = 0 \\ \kappa V_\Psi - b_r e^{(-\lambda P_{\Phi k})} V_{\Phi k} - \mu_r V_{\Phi k} = 0 \\ b e^{-\lambda P_{\Phi k}}[R_{\Phi k} + V_{\Phi k}] - \mu C_{\Phi k} = 0 \\ aC_{\Phi k} - bS_{\Phi k} = 0 \\ bS_{\Phi k} - bR_{\Phi k} = 0 \\ a_P C_{\Phi k} - b_p P_{\Phi k} = 0 \end{cases} \Rightarrow \begin{cases} V_\Psi = \frac{b(1-e^{-\lambda P_{\Phi k}})}{c+\kappa} R_{\Phi k} \\ P_\Psi = \frac{b_p}{c} P_{\Phi k} \\ V_{\Phi k} = \frac{\kappa b(1-e^{-\lambda P_{\Phi k}})}{(c+\kappa)(b_r e^{(-\lambda P_{\Phi k})}+\mu_r)} R_{\Phi k} \\ C_{\Phi k} = \frac{b e^{-\lambda P_{\Phi k}}}{\mu}[1 + \frac{\kappa b(1-e^{-\lambda P_{\Phi k}})}{(c+\kappa)(b_r e^{(-\lambda P_{\Phi k})}+\mu_r)}] R_{\Phi k} \\ S_{\Phi k} = \frac{a}{b} C_{\Phi k} \\ R_{\Phi k} = S_{\Phi k} \\ P_{\Phi k} = \frac{a_P}{b_p} C_{\Phi k} \end{cases}$$

For this to be consistent as before, we need $\frac{b e^{-\lambda P_{\Phi k}}}{\mu}[1 + \frac{\kappa b(1-e^{-\lambda P_{\Phi k}})}{(c+\kappa)(b_r e^{(-\lambda P_{\Phi k})}+\mu_r)}] = \frac{b}{a}$ so that we have $C_{\Phi k} = \frac{b}{a} R_{\Phi k} = \frac{b}{a} S_{\Phi k} = C_{\Phi k}$.

$$\begin{aligned} & \frac{b e^{-\lambda P_{\Phi k}}}{\mu}[1 + \frac{\kappa b(1-e^{-\lambda P_{\Phi k}})}{(c+\kappa)(b_r e^{(-\lambda P_{\Phi k})}+\mu_r)}] = \frac{b}{a} \\ \Leftrightarrow \quad & cbe^{-2\lambda P_{\Phi k}} + ((c+\kappa)\mu_r - \frac{(c+\kappa)b_r\mu}{a} + \kappa b)e^{-\lambda P_{\Phi k}} = \frac{\mu(c+\kappa)\mu_r}{a} \\ \Leftrightarrow \quad & Ae^{-2\lambda P_{\Phi k}} + Be^{-\lambda P_{\Phi k}} + C = 0 \end{aligned}$$

Let us set $e^{-\lambda P_{\Phi k}} = X$ then we need to solve $AX^2 + BX + C = 0$:

$$\Delta = B^2 - 4AC > 0 (\text{computing values}) \Rightarrow X_{1,2} = \frac{-B \mp \sqrt{\Delta}}{2A}$$

And finally we found $e^{-\lambda P_{\Phi k}} = X_1$ leading to $P_{\Phi k} = \frac{1}{\lambda}\log(X_1)$ and so that $C_{\Phi k} = \frac{b_p}{a_P} P_{\Phi k} = \frac{b_p}{a_P \lambda}\log(X_1)$.

With parameters values set as in Table 2, Table of Parameters, we should have (in copies.cell$^{-1}$ or copies.mL$^{-1}$ in Sinusoids):

$$
\begin{aligned}
V_\Psi &\approx 128.3\\
P_\Psi &\approx 1.376e+05\\
V_{\Phi k} &\approx 55.5\\
C_{\Phi k} &\approx 11.44\\
S_{\Phi k} &\approx 825.6\\
R_{\Phi k} &\approx 825.6\\
P_{\Phi k} &\approx 8.256e+5
\end{aligned}
$$

Thanks to the strong law of large numbers, we can assume that the agent-based part of the model does not affect the steady state too much.

We can also observe the individual impact of non-CYL immune response with strength $u$ as it is only a term added to equations above.

Simulation in the homogeneous base line case results in a steady state where $C_{\Phi k}$ $R_{\Phi k}$ $S_{\Phi k}$ $P_{\Phi k}$ are really close to this analysis (relative error $< 1\%$). However for $V_\Psi$, $V_{\Phi k}$ and $P_\Psi$ the results differ from those found in the analysis above as these particles are the most affected by the other parts of the model (circulation to other subcompartments for $V_\Psi$ and $P_\Psi$, and $V_{\Phi k}$ is directly linked to the presence of $V_\Psi$). This steady state analysis was thus a good way to verify good or bad implementations during the construction of the numerical model.
